# Supplementary material for: Development and Validation of Quality Indicators for Pulmonary Arterial Hypertension Management in Japan: A Modified Delphi Consensus Study
Source: Diagnostics (Basel). 2024 Nov 25;14(23):2656. doi: 10.3390/diagnostics14232656 (PMC11640398; doi:10.3390/diagnostics14232656)
Supplement: Supplementary file 1 [file diagnostics-14-02656-s001.zip › diagnostics-3324090-supplementary.pdf]

Supplemental Table S1: Initial version of quality indicators for the care and outcomes of adults with pulmonary arterial hypertension

Domain	Content	Med	Min	Max	Mode
**1. Structural framework**					
**1.1**	Pulmonary hypertension centers that have a specialized MDT responsible for the management of patients with PAH<br>**Note:** MDT consists of at least a cardiologist, pulmonologist, and specialist nurse. Collaborations should be established with a rheumatologist, interventional radiologist, cardiothoracic surgeon, social worker, and psychologist.	9	7	9	9
**1.2**	Pulmonary hypertension centers that have the following facilities and skills:				
	- a ward where healthcare providers have expertise in PAH;	9	9	9	9
	- a specialist outpatient service;	9	8	9	9
	- an intermediate/intensive care unit;	9	8	9	9
	- a 24/7 emergency care;	9	8	9	9
	- an interventional radiology unit (for treatment of hemoptysis);	8	6	9	7
	- diagnostic investigations, including echocardiography, CT scanning, nuclear medicine, MRI, exercise tests, and PFT;	9	9	9	9
	- a cardiac catheterization laboratory with vasodilator testing available;	9	3	9	9
	- an access to genetic counseling and testing;	8	1	9	9
	- fast and easy access to cardiothoracic and vascular surgery, cardiac anesthesia, and ECMO	9	7	9	9
	- established collaboration with a lung/heart–lung transplantation center	9	8	9	9
**1.3**	Pulmonary hypertension centers that participate in a national or an international PAH registry	9	7	9	9
**1.4**	Pulmonary hypertension centers that have a fast-track policy to review urgent referrals within 1–2 weeks	8	7	9	9
**2. Diagnosis and risk stratification**					
**2.1**	Proportion of patients with suspected PAH who undergo pulmonary function test (including lung volumes and DLCO) at the time of diagnostic work-up	9	8	9	9
**2.2**	Proportion of patients with suspected PAH who have echocardiography at the time of diagnostic work-up	9	9	9	9

**2.3**	Proportion of patients with suspected PAH who have a RHC at the time of diagnostic work-up	9	9	9	9
**2.4**	Proportion of patients with suspected PAH who have perfusion imaging (V/Q scan or new modality) to exclude CTEPH at the time of diagnostic work-up<br>**Note:** Alternative perfusion imaging techniques include iodine subtraction mapping, dual-energy CT, or MRI perfusion	9	9	9	9
**2.5**	Proportion of patients with suspected PAH who have been screened for CTD at the time of diagnostic work-up	9	8	9	9
**2.6**	Proportion of patients with a diagnosis of idiopathic, heritable, or drug-induced PAH who have RHC with acute vasodilator testing at the time of diagnostic work-up	9	3	9	9
**2.7**	Proportion of patients with a diagnosis of PAH who have their WHO-FC, NT-proBNP and 6MWT assessed at the time of PAH diagnosis	9	6	9	9
**2.8**	Proportion of patients with a diagnosis of PAH who have their risk assessed using a validated tool (e.g. ESC/ERS guidelines) at the time of PAH diagnosis	9	5	9	9
**2.9**	Proportion of patients with a diagnosis of PAH who have their quality of life assessed using a validated tool (Emphasis-10, SF-36, etc.) at the time of PAH diagnosis	7	1	9	7
**2.10**	Pulmonary hypertension centers that can perform exercise right heart catheterization for patients with suspected PAH who are at high risk at the time of diagnostic work-up	8	2	9	9
**3. Initial treatment**					
**3.1**	Proportion of patients with a diagnosis of non-vasoreactive idiopathic, heritable, or drug-associated PAH and at high risk without significant cardiopulmonary comorbidities who are prescribed i.v./s.c. prostacyclin analogues	8	7	9	9
**3.2**	Proportion of patients with a diagnosis of non-vasoreactive idiopathic, heritable, drug-associated or CTD-associated PAH and at low or intermediate risk without significant cardiopulmonary comorbidities who are prescribed initial combination therapy with a NO donor and an ERA	9	6	9	9
**3.3**	Proportion of patients with a diagnosis of vasoreactive idiopathic, heritable, or drug-associated PAH and acute vasodilator response who are prescribed high doses of calcium channel blockers	6	1	9	6
**4. Follow-up**					

**4.1**	Proportion of patients with a diagnosis of PAH who have their risk assessed using a validated tool (e.g. ESC/ERS guidelines) at least every 6 months	8	6	9	9
**4.2**	Proportion of patients with a diagnosis of PAH who have been informed about available patient association/support group(s)	7	5	9	8
**4.3**	Proportion of patients with a diagnosis of PAH who have their WHO-FC, NT-proBNP and 6MWT assessed at least every 6 months	8	3	9	9
**4.4**	Proportion of patients with a diagnosis of PAH in whom low risk is not achieved who have a discussion with a member of the MDT on treatment strategy	7	3	9	7
**4.5**	Proportion of patients with a diagnosis of PAH and at intermediate-high or high risk who are evaluated for lung transplantation<br>**Note:** Who are eligible for lung transplantation (based on age and comorbidities) and have been established on a combination therapy.	8	6	9	8
**4.6**	Proportion of patients with a diagnosis of PAH in whom regular hemodynamic assessment is considered at least every 12 months	9	7	9	9
**Secondary 4**	Proportion of patients with a diagnosis of PAH who have their quality of life assessed using a validated tool at least every 6 months	7	1	9	7
**5. Outcomes**					
**5.1**	Median time between establishing the diagnosis of PAH (i.e. date of diagnostic RHC) and commencing PAH therapy	9	1	9	9
**5.2**	Median time between referral and commencing PAH therapy<br>**Note:** Referral time is date of the receipt by the specialist PAH center of the referral request	9	7	9	9

Med: median, Min: minimum, Max: maximum, Mo: mode

PAH: pulmonary arterial hypertension, MDT: multidisciplinary team, CT: computed tomography, MRI: magnetic resonance imaging, PFT: pulmonary function test, ECMO: extracorporeal membrane oxygenation, RHC: right heart catheterization, CTEPH: chronic thromboembolic pulmonary hypertension, DLCO: diffusing capacity of the lung for carbon monoxide, CTD: connective tissue disease, WHO-FC: World Health Organization functional class, NT-proBNP: N-terminal pro B-type natriuretic peptide, BNP: B-type natriuretic peptide, 6MWT: 6-Minute Walk Test, ESC/ERS: European Society of Cardiology/European Respiratory Society, SF-36, 36-Item Short Form Health Survey, i.v.: intravenous, s.c.: subcutaneous, NO: nitric oxide, ERA: endothelin receptor antagonist

Sup Table S2: The Japanese final version of quality indicators for the care and outcomes of adults with pulmonary arterial hypertension

ドメイン	内容
**1. 医療機関の枠組み**	
**1.1**	PAH 患者の管理を担当する専門的な多診療科・多職種チーム(MDT)を有する肺高血圧症診療機関である<br>（MDT は少なくとも循環器専門医・呼吸器専門医・専門看護師で構成される。リウマチ専門医、インターベンショナルラジオロジスト、心臓・胸部外科医、ソーシャルワーカー、心理学者とは協力体制を確立する必要がある)
**1.2**	肺高血圧症専門診療機関は、以下の施設とスキルを有する<br>- PAH に精通した医療従事者が病棟管理を実施できる<br>- 肺高血圧症の専門外来を設けている<br>- 肺高血圧症に対応できる集中治療室が存在する<br>- 24 時間体制の救急医療を提供できる<br>- 喀血の治療に対応できるインターベンショナル・ラジオロジー・ユニットが存在する<br>- 心エコー、CT スキャン、核医学、MRI、運動負荷試験、呼吸機能検査などの診断的検査が実施できる<br>- 心臓カテーテル検査で急性血管反応試験が実施できる<br>- 遺伝カウンセリングと遺伝子検査を受けることが出来る<br>- 心臓外科、血管外科、心臓麻酔、ECMO への迅速かつ容易にアクセスができる<br>- 肺移植・心肺移植センターとの連携が確立されている
**1.3**	国内または国際的な PAH レジストリに参加している
**1.4**	緊急の紹介患者に対して 1～2 週間以内にアセスメントを完了する迅速な対応トラックを有する
**2. 診断とリスク層別化**	
**2.1**	PAH 疑い患者に対する診断ワークアップ時に呼吸器能検査（肺活量と DLCO を含む）を行った割合
**2.2**	PAH 疑い患者に対する診断ワークアップ時に心エコー検査を行った割合
**2.3**	PAH 疑い患者に対する診断ワークアップ時に右心カテーテル検査を行った割合
**2.4**	PAH 疑い患者に対する診断ワークアップ時に CTEPH を除外するために肺血流画像検査（V/Q スキャンまたは新しいモダリティ）を行った割合
**2.5**	PAH 疑い患者に対する診断ワークアップ時に結合組織病（CTD）のスクリーニングを行った割合
**2.6**	特発性、遺伝性、薬剤性 PAH と診断された患者のうち、診断時に急性血管性拡張検査を含めた右心カテーテル検査を実施した割合

**2.7**	PAH と診断された患者のうち、PAH 診断時に WHO-FC、NT-pro BNP（/BNP）、6MWT の評価を行った患者の割合
**2.8**	PAH と診断された患者のうち、PAH 診断時に有効なリスク層別化ツール（ESC/ERS ガイドラインなど）を用いてリスク評価を行った患者の割合
**2.9**	PAH と診断された患者のうち PAH 診断時に日本語でバリデーションされたツール（emPHasis-10,SF-36 など）を用いて QOL 評価をした患者の割合
**Secondary 2**	診断ワークアップ時に PAH を発症するリスクが高い症例に対して運動負荷右心カテーテル検査の実施ができる
**3. 初期治療**	
**3.1**	特発性、遺伝性、または薬物関連 PAH の診断を受け、リスク評価で高リスクと判定され、重要な心肺の合併症や急性血管反応試験も陰性である患者の中で、静脈内/皮下のプロスタサイクリン・アナログが処方される患者の割合
**3.2**	リスク評価で低リスクまたは中等度のリスクと判定され、重要な心肺の合併症がなく、急性血管反応試験も陰性の特発性、遺伝性、薬物関連、または結合組織病関連 PAH の診断を受けた患者で、NO 系薬剤と ERA の組み合わせで upfront 併用療法が処方される患者の割合
**3.3**	急性血管拡張反応を示す血管反応性の PAH 患者（特発性、遺伝性、または薬物関連 PAH）の中で、高用量カルシウム拮抗薬が処方された患者の割合
**4.フォローアップ**	
**4.1**	PAH と診断された患者のうち少なくとも 6 ヵ月に 1 度、有効なリスク層別化ツール（ESC/ERS ガイドラインなど）を用いてリスク評価を受けている患者の割合
**4.2**	PAH の診断を受けた患者で、利用可能な患者会/支援団体について情報提供を行う患者の割合
**4.3**	PAH と診断された患者のうち少なくとも 6 ヶ月に 1 度、WHO-FC、NT-pro BNP もしくは BNP、6MWT の評価を行っている患者の割合
**4.4**	PAH と診断された患者でリスク評価で低リスクが達成されない場合に、治療方針について多診療科・多職種チーム（MDT）のメンバーでカンファレンスを行った患者の割合
**4.5**	PAH と診断され中高リスクまたは高リスク患者における肺移植の評価が行われる患者の割合（年齢、併存疾患に基づく評価に基づく肺移植の適応があり、併用療法が確立されている人を母数とする）
**4.6**	PAH と診断された患者のうち低リスクではない患者に対して少なくとも 12 ヵ月に 1 度、定期的な血行動態評価の実施を検討している患者の割合
**Secondary 4**	PAH と診断された患者のうち少なくとも 6 ヵ月に 1 度、有効なツールを用いて QOL を評価した患者の割合
**5. アウトカム**	

**5.1**	PAH の診断確定（カテーテル実施日）から PAH 治療を開始するまでの期間（日数の中央値）
**5.2**	PH 診療施設に紹介を受けて（紹介連絡日）から PAH 治療を開始するまでの期間（日数の中央値）
